# Effective coverage of essential antenatal care interventions: A cross-sectional study of public primary healthcare clinics in the West Bank

**DOI:** 10.1371/journal.pone.0212635

**Published:** 2019-02-22

**Authors:** Mahima Venkateswaran, Binyam Bogale, Khadija Abu Khader, Tamara Awwad, Ingrid K. Friberg, Buthaina Ghanem, Taghreed Hijaz, Kjersti Mørkrid, J. Frederik Frøen

**Affiliations:** 1 Global Health Cluster, Division for Health Services, Norwegian Institute of Public Health, Oslo, Norway; 2 Centre for Intervention Science in Maternal and Child Health (CISMAC), University of Bergen, Bergen, Norway; 3 Palestinian National Institute of Public Health, World Health Organization, Ramallah, Palestine; 4 Ministry of Health, Ramallah, Palestine; Faculty of Medicine & Dalla Lana School of Public Health, CANADA

## Abstract

**Background:**

The proportion of women attending four or more antenatal care (ANC) visits is widely used for monitoring, but provides limited information on quality of care. Effective coverage metrics, assessing if ANC interventions are completely delivered, can identify critical gaps in healthcare service delivery. We aimed to measure coverage of at least one screening and effective coverage of ANC interventions in the public health system in the West Bank, Palestine, and to explore associations between infrastructure-related and maternal sociodemographic variables and effective coverage.

**Methods:**

We used data from paper-based clinical records of 1369 pregnant women attending ANC in 17 primary healthcare clinics. Infrastructure-related variables were derived from a 2014 national inventory assessment of clinics. Sample size calculations were made to detect effective coverage ranging 40–60% with a 2–3% margin of error, clinics were selected by probability sampling. We calculated inverse probability weighted percentages of: effective coverage of appropriate number and timing of screenings of ANC interventions; and coverage of at least one screening.

**Results:**

Coverage of one screening and effective coverage of ANC interventions were notably different for screening for: hypertension (98% vs. 10%); fetal growth abnormalities (66% vs. 6%); anemia (93% vs. 14%); gestational diabetes (93% vs. 34%), and antenatal ultrasound (74% vs. 24%). Clinics with a laboratory and ultrasound generally performed better in terms of effective coverage, and maternal sociodemographic factors had no associations with effective coverage estimates. Only 13% of the women attended ANC visits according to the recommended national schedule, driving effective coverage down.

**Conclusion:**

Indicators for ANC monitoring and their definitions can have important consequences for quantifying health system performance and identifying issues with care provision. To achieve more effective coverage in public primary care clinics in the West Bank, efforts should be made to improve care provision according to prescribed guidelines.

## Introduction

Antenatal care (ANC) provides an opportunity to detect risk factors, prevent complications and improve birth preparedness of pregnant women in order to reduce maternal and neonatal morbidity [1, 2]. The proportion of women who attend four or more ANC visits (ANC 4+), is used extensively as an indicator for monitoring health of pregnant women as well as health system performance [3, 4]. However, measuring contact of pregnant women with the health system has limitations, since attending an ANC visit does not imply that pregnant women receive good quality care [5–7]. The quality of care received may also be inequitable. In low and middle-income countries (LMIC), even with high levels of ANC 4+, wealthier and better-educated women are significantly more likely to receive quality care [8].

Effective coverage, in contrast, combines utilization of healthcare services with the quality of care received. Conceptually, effective coverage is “the proportion of the population who need a service that receive it with sufficient quality for it to be effective” [9]. For ANC, effective coverage is conventionally comprised of ‘ANC attendance’, defined as having at least one or at least four ANC visits; and ‘quality’, assessed in terms of ANC content [10]. Standard ANC content includes a set of interventions, which entail single, two-step or repeat screening tests and managements at specified times during pregnancy [11, 12]. The World Health Organization has published widely accepted recommendations for ANC [13], including suggestions for appropriate contact (frequency and timing between clients and the health system) and content (screening and management) based on evidence of effectiveness [14, 15].

Whether pregnant women have received some or all components of a set of interventions as part of ANC at least once during pregnancy has been used to indicate quality of care [9, 16, 17]. This measure, without timing or frequency, is not adequate to measure effectiveness or quality of care provided. For example, one hemoglobin measurement in pregnancy does not correspond to the provision of effective interventions for prevention and management of anemia as recommended by the WHO guidelines–being tested only late in pregnancy excludes the opportunity for treatment, and being tested only early does not imply a safe hemoglobin level at delivery. Measuring effective coverage of essential ANC interventions is, therefore, more comprehensive than ANC4+ for assessing ANC service provision [10].

Assessing effective coverage can help identify critical ‘bottlenecks’ around provision of healthcare such as care providers’ knowledge of clinical practice guidelines and infrastructure availability [18, 19]. Typical health systems ‘bottlenecks’, which limit its capacity to provide effective care, include access to care, availability of trained human resources and health infrastructure as well as utilization [20]. Studies assessing ANC content and quality in LMIC often use population-based surveys as the main data source. In general, household surveys provide limited information on processes of care and the accuracy of information collected is reliant on recall of survey participants [21]. Facility-based documentation and direct observations [22] can be used to assess effective coverage of ANC interventions at a given visit. Facility-based data, if available routinely over a period of time, can provide information on the number and timing of screening tests of ANC interventions provided–aspects of healthcare provision not available from household surveys [23, 24].

Better health information systems and improving the quality of healthcare services are of high priority for the Palestinian health system [25, 26], with no published studies of health system performance or ANC provision in public primary healthcare clinics in the West Bank available. In the West Bank, maternal and child health services are organized in two tiers–primary healthcare where ANC, postpartum care and newborn care are provided; and secondary or tertiary healthcare where obstetric services are provided. The public sector is reportedly the single largest provider of ANC, catering to almost 50% of all women that give birth in a year [27]. Based on place of residence, pregnant women are assigned to a governmental primary healthcare clinic for care. ANC is also provided by private health facilities, non-governmental organizations and the United Nations Relief and Works Agency for Palestine Refugees in the Near East (UNRWA) [27]. A recent household survey suggests that more than 95% of women attend 4 or more ANC visits [28]. The Palestinian Ministry of Health and the Palestinian National Institute of Public Health are currently implementing an electronic health information system for maternal and child health consisting of individual-level data collected at the point-of-care (eRegistry) in public primary healthcare clinics [29]. As a result of this implementation, the existing data ecosystem for maternal and child health is shifting from aggregated data on the mean number of ANC visits per pregnant woman to individual-level data with accessible information on content and processes of ANC service delivery. Such a transition could be disruptive to the health system if the nature and magnitude of any changes to the available data and indicators, and associated factors are not anticipated or not understood by health system managers.

In this study, our objective was to assess the coverage of at least one screening and appropriate number of screenings of ANC interventions, and effective coverage of ANC interventions in public primary healthcare clinics in the West Bank, Palestine. Secondarily, we explored selected infrastructure-related and maternal sociodemographic factors potentially associated with effective coverage.

## Materials and methods

We extracted data from paper-based clinical records of antenatal care to demonstrate the potential changes in health and health systems performance indicators that would be observed when transitioning from the existing aggregate health information system to the eRegistry. Since the Palestinian national eRegistry implementation was rolled out in phases, we extracted records from a random cross-sectional sample of clinics in the five districts that comprised phase one, from the year 2015, before any clinics started using the eRegistry.

### Study setting

ANC records (paper-based until 2016 and the eRegistry thereafter) are primarily used for clinical documentation in all primary healthcare clinics. Paper-based ANC records were structured data entry forms consisting of data elements pertaining to clients’ medical history, screening tests results, clinical examinations, and clinical managements [29]. While nurses or midwives typically provide routine ANC in primary healthcare clinics, doctors visit the clinic once or twice a week and perform clinical and ultrasound examinations and interpret lab test results, and manage complications in pregnancies. Clinics may either have their own laboratory and ultrasound or share these facilities with other clinics.

### Sample size and sampling

A single data collection exercise was set up to measure maternal morbidity rates [30] as well as effective coverage of ANC interventions. The overall sample size was determined by the least prevalent outcome expected, corresponding to a 1% prevalence of severe anemia in pregnancy. It was assumed that effective coverage of ANC interventions would be in the 40–60% range (based on expert opinion in the absence of relevant data). In order to estimate indicators in these ranges that were representative of the five phase 1 districts, and with margins of error of 2–3% for the coverage of ANC interventions and 0.5% for maternal morbidity rates, 1344 pregnancies were required [31]. OpenEpi was used for sample size calculations [31].

Primary healthcare clinics were selected by probability sampling proportional to clinic size until a sufficient number of clinics was sampled to achieve the calculated sample size (n = 1344), provided that clinical records of all women registered for ANC in these clinics during January–December 2015 were included in the data collection. Since the primary healthcare clinics were selected by unequal probability sampling, inverse probability sample weights were assigned to individual pregnancies in order to produce results that were more generalizable to the five districts included in the data collection, and to produce robust standard errors [32]. The same dataset was used for the calculation of prevalences of maternal health conditions and details are presented elsewhere [30].

### Data collection

Data were extracted from paper-based ANC records and entered into electronic forms on the District Health Information System 2 (DHIS2) software, which were identical to the data capture forms of the eRegistry, to ensure similar data structures [33]. Two trained data collectors, who were nurse-midwives, extracted data from clinical records. Ten per cent of the clinical records were extracted and entered twice by each of the data collectors and the study team carried out consistency checks of the double-entered data [34].

An inventory assessment of all public primary healthcare clinics in the West Bank was completed by the study team at the Palestinian National Institute of Public Health in December 2014. Information needed to support the implementation of the eRegistry was collected, including details of infrastructure in the clinics, laboratory and ultrasound availability, and the number and type of care providers for maternal and child health [29, 34]. Clinic staff were asked to return completed assessment forms to the study team; 100% of clinics completed this form.

### Outcome variables

ANC interventions included in our analyses comprised those that were: 1) recommended as part of routine ANC content in the public health system in the West Bank; 2) applicable to all pregnant women irrespective of risk status; and 3) amenable to measurement using data from ANC records. Applying these criteria, eight ANC interventions were selected (Table 1). Three of these interventions were similar to the WHO Essential interventions [35], and four of the interventions were recommended as part of the WHO ANC model for a positive pregnancy experience [13] (Table 1). Six additional ANC interventions recommended in the public health system were excluded from this analysis, either because the ANC records did not contain the variables required to generate the indicators or because the interventions were not appropriate for the primary healthcare level (S1 Text).

**Table 1:** Recommended schedule of ANC visits and ANC interventions in the West Bank.

ANC interventions	Recommended ANC visits schedule				
	Booking^ᵻᵻ^	16 weeks	24–28 weeks	32 weeks	36 weeks
Screening for hypertension*	**X**	**X**	**X**	**X**	**X**
**SFH measurement^¥^**	**X**	**X**	**X**	**X**	**X**
Screening for anemia*	**X**		**X**		**X**
**Antenatal ultrasound^§^**	**X**		**X**		**X**
**Screening for gestational diabetes mellitus^¥^**	**X** **(Urine)**		**X** **(Blood)**		
**Screening for asymptomatic bacteriuria^¥^**	**X**				
**Screening for Rh-type^¥^**	**X**				
Screening for tetanus immunization status*	**X**				

*Similar to the WHO’s Essential Interventions for RMNCH

^¥^Recommended in the 2016 WHO ANC model for a positive pregnancy experience

^§^Context-specific recommendation

^ᵻᵻ^Booking: refers to first antenatal visit at the clinic; ANC: Antenatal care; SFH: Symphysis-fundal height.

For each ANC intervention selected, we defined indicators of coverage of at least one screening test, coverage of appropriate number of screenings (only applicable to ANC interventions requiring repeat or two-step screening), and effective coverage, based on ANC guideline in the West Bank (Table 2). Definitions for effective coverage of ANC interventions included both the recommended timing and number of screening tests of the intervention (Table 2).

**Table 2:** Definitions of indicators of coverage of at least one screening, coverage of appropriate number of screenings, and effective coverage of ANC interventions.

ANC intervention	Coverage of at least 1 screening	Coverage of the appropriate number of screening	Effective coverage (appropriate number and timing of screenings)
**Screening for hypertension**	Proportion with at least one blood pressure measurement	Proportion with five blood pressure measurements	Proportion with blood pressure measurements at all recommended ANC visits
**SFH measurement**	Proportion with at least one SFH measurement	Proportion with five SFH measurements	Proportion with SFH measured at all recommended ANC visits^ᵻ^
**Screening for anemia**	Proportion with at least one hemoglobin test	Proportion with three hemoglobin tests	Proportion with hemoglobin tests at booking^ᵻᵻ^, 24–28 and 36 weeks*
**Antenatal ultrasound**	Proportion with at least one ultrasound examination	Proportion with three ultrasound examinations	Proportion with ultrasound examinations at booking^ᵻᵻ^, 24–28 and 36 weeks*
**Screening for gestational diabetes mellitus**	Proportion with either urine sugar or blood sugar test	Proportion with both urine sugar and blood sugar test	Proportion with urine sugar test at booking^ᵻᵻ^ and blood sugar test at 24–28 weeks*
**Screening for asymptomatic bacteriuria**	Proportion with urine microscopy test		Proportion with urine microscopy test at booking^ᵻᵻ^
**Screening for Rh-type**	Proportion with Rh-typing		Proportion with Rh-typing at booking visit
**Screening for tetanus immunization status**	Proportion whose tetanus immunization status is checked by asking for history of immunization or reviewing immunization record		Proportion whose tetanus immunization is checked by asking for history of immunization or reviewing immunization record at booking^ᵻᵻ^

^ᵻ^calculated for ANC visits that occur after 16 weeks

*given that registration of pregnancy was before the recommended timing of screening

^ᵻᵻ^Booking: refers to first antenatal visit at the clinic.

ANC: Antenatal care; SFH: Symphysis-fundal height

In the definitions for effective coverage of ANC interventions, the appropriate number of timely screening tests were adjusted according to the gestational age of pregnant women at registration for ANC. For example, women who were registered for ANC before 24 gestational weeks were considered effectively screened for anemia if they had three hemoglobin tests–at first ANC visit, at 24–28 weeks and 36 weeks (Table 2), while women that were registered for ANC after 28 weeks were considered effectively screened if they received two hemoglobin tests, one at their first ANC visit and another at 36 weeks (Table 2).

We calculated the proportion of women with any four and any five ANC visits irrespective of timing of visits. Since coverage of appropriate number of screening tests and effective coverage are influenced by attendance rates following pregnancy registration, we calculated the proportion attending all timely visits appropriate to when the first ANC visit occurs. We measured the proportion of women attending ANC visits in the specific time windows where interventions were recommended (Table 1). We also assessed the proportion attending all 5 timely visits including an early first ANC visit before 14 weeks.

### Variables potentially associated with effective coverage

Laboratory and ultrasound availability were the infrastructure-related factors chosen for analyses, since these were expected to be associated with effective coverage. Clinics were grouped into those that had all relevant infrastructure and those that had one or more missing infrastructure. Since the sample of clinics had similar cadres of care providers, and were expected to be similar in terms of availability of other infrastructure needed for ANC (e.g. sphygmomanometers), we did not use these for exploratory analyses.

Maternal sociodemographic variables used in the analyses were those available in the ANC records, including women’s age at pregnancy registration, age at marriage, education and parity.

### Data analyses

All analyses were done using STATA version 15 (StataCorp. 2015. *Stata Statistical Software*: *Release 14*. College Station, TX: StataCorp LP), using the command ‘svyset’ for generating weighted proportions and 95% confidence intervals (CI) [36]. Descriptive statistics were produced for the following variables and categories: women’s age at pregnancy registration (<21 years, 21–34 years, >34 years); age at marriage (<20 years and ≥20 years); number of years of education of women (<10 years, 10–13 years, >13 years); and parity (nulliparous, multiparous<4, multiparous≥4). These categories were pre-defined in the dataset obtained for this analysis in accordance with the data sharing policies outlined in the Standard Operating Procedures for routine registry operations [34].

Chi-square tests of differences were used for exploratory analyses of effective coverage of ANC interventions across sub-groups based on infrastructure-related and maternal sociodemographic variables. Adjusted odds ratios (OR) and 95% CI were generated for each of the interventions, through a logistic regression model consisting of infrastructure-related characteristics (laboratory and ultrasound availability) and all maternal sociodemographic variables (women’s age at pregnancy registration, education, age at marriage and parity).

### Ethics approval

Anonymous secondary data for analyses were obtained with approvals from the Palestinian Ministry of Health, in accordance with the data sharing principles outlined in the Standard Operating Procedures for routine registry operations [34]. Ethics approvals for this study were obtained from the Palestinian Health Research Council (PHRC/HC/272/17) and the Regional Committee for Medical and Health Research Ethics in Norway (2017/1537). Descriptions to re-create identical data, as well as contact addresses to the data source, are available as supporting information (S2 Text).

## Results

Data were collected from 1369 clinical records of pregnant women first registered for ANC in 2015 in 17 primary healthcare clinics. Totally, these women attended 6397 ANC visits during 2015 and 2016. One out of the 17 primary healthcare clinics had a non-nurse/midwife health worker that was the sole provider of ANC, while all other clinics had a nurse or midwife providing ANC. All 17 clinics had a doctor visiting once a week to provide ANC. Of the 17 primary healthcare clinics, six were equipped with both a laboratory and ultrasound. Two clinics each had either only a laboratory or only an ultrasound, while seven clinics had neither.

Fifty-four pregnancies in the sample (4%) ended in a documented spontaneous miscarriage. The mean gestational age at first ANC visit was 14 weeks (SD = 7), 47% of the women (95% CI: 38, 55, n = 638) attended their first ANC visit within 3 months and 67% of women (95% CI: 60, 73, n = 914) attended their first ANC visit within 4 months. The majority (75%) of women were between 21–35 years of age at the time of their first ANC visit, and 37% were nulliparous (Table 3).

**Table 3:** Background sociodemographic characteristics of pregnant women in the sample.

Sociodemographic characteristics	Population (n)	Percentage
Age		
*<20*	222	16
*21–35*	1029	75
*>35*	118	9
Education		
*<10*	149	11
*10–13*	591	43
*>13*	514	37
Age at marriage		
*<20*	695	50
*>20*	573	42
Parity		
*Nulliparous*	501	37
*Multiparous (<4)*	666	48
*Multiparous (≥4)*	186	14

### ANC attendance

About half of the women attended at least five ANC visits, while 60% (95% CI: 50, 70) attended at least four ANC visits, when not considering the schedule or timing of visits (Table 4). Only 6% (95% CI: 5, 8) of the women attended all ANC visits according to the recommended ANC 5-visit schedule, including an early first ANC visit before 16 weeks. Disregarding early attendance and only considering the schedule of visits after pregnancy registration, 13% (95% CI: 9, 17) attended ANC visits as per the recommended national schedule (Table 4), and thus could have received complete hypertension and SFH screening.

**Table 4:** Comparison of coverage at least one screening of ANC intervention, coverage of appropriate number of screenings prescribed for ANC interventions, and effective coverage of ANC interventions (number and timing of screening of ANC interventions).

ANC intervention	Coverage of ANC interventions^§^ (%, 95% CI)			ANC visits *(%*, *95% CI)*	
	At least one screening test	Appropriate number of screening tests	Effective coverage	*Number of visits irrespective of timing*^*‡*^	*Appropriate number and timing of visits*^*‡*^
Screening for hypertension	**98 (96, 99)**	**38 (31, 47)**	**10 (8, 13)**	*48 (38*, *58)*	*13 (9*, *17)*
SFH measurement	**66 (50, 80)**	**35 (24, 48)**	**6 (4, 9)**		
Screening for anemia	**93 (89, 96)**	**31 (23, 40)**	**14 (9, 21)**	*73 (62*, *81)*	*33 (26*, *41)*
Antenatal ultrasound	**74 (59, 85)**	**43 (32, 54)**	**24 (18, 31)**		
Screening for gestational diabetes mellitus	**93 (88, 96)**	**69 (60, 77)**	**34 (26, 43)**	*85 (77*, *90)*	*56 (50*, *62)*
Screening for asymptomatic bacteriuria*	**55 (45, 64)**		**42 (36, 49)**^**¥**^	*NA*	
Screening for Rh-type*	**78 (67, 89)**		**64 (54, 73)**^**¥**^	*NA*	
Screening for tetanus immunization status*	**35 (23, 50)**			*NA*	

^§^refer Table 2 for definitions of coverage indicators of ANC interventions

^‡^refer Table 1 for number of ANC visits and their timing for each ANC intervention recommended in the national guidelines

*only one screening test during ANC is recommended in the national guidelines

^**¥**^refers to screening test provided during the first ANC visit.

ANC: Antenatal Care; SFH: Symphysis-fundal height; CI: Confidence Intervals

The proportion of women attending all recommended ANC visits according to the national guidelines was higher in clinics with both laboratory and ultrasound (17%), compared to clinics with one or no such infrastructure (9%), with an adjusted OR of 2.0 (95% CI: 1.4, 2.8).

### Coverage of ANC interventions

Coverage of at least one sreening of ANC interventions ranged between 55% (95% CI: 45, 64) for screening for asymptomatic bacteriuria and 98% (95% CI: 96, 99) for hypertension screening (Table 4).

Compared to the coverage of at least one screening, coverage of the appropriate number of screenings was considerably lower for all interventions requiring repeat or two-step screening (Table 4). In clinics that had ultrasound equipment, coverage of any symphysis fundus height (SFH) measurement was 29%, while in clinics without ultrasound the coverage was 63%.

For diabetes screening, coverage of blood sugar test was 73% (95% CI: 65, 79) and urine sugar test was 89% (95% CI: 82, 94).

### Effective coverage

Effective coverage of ANC interventions was lower than the coverage of at least one screening and coverage of appropriate number of screenings for all interventions except screening for tetanus immunization status (Table 4). Regarding screening for gestational diabetes mellitus, 43% (95% CI: 35, 52) had a blood sugar test at 24–28 weeks and 71% (95% CI: 63, 78) had a urine sugar test at booking visit.

Among those attending the prescribed number and timing of ANC visits (Table 4), the percentage receiving the relevant screening tests were as follows: hypertension screening: 77%, antenatal ultrasound: 73%, gestational diabetes: 61%, SFH measurement: 46% and anemia screening: 42%.

Effective coverage of six of the eight ANC interventions was highest in primary healthcare clinics with laboratory and ultrasound availability (Table 5). Clinics with a laboratory and ultrasound were associated with statistically significant higher odds of effectively screening for four ANC interventions. Screening for tetanus immunization status was the only ANC intervention that had a statistically significant lower odds ratio (adjusted OR = 0.7, 95% CI: 0.5, 0.9) (Table 5).

**Table 5:** ANC interventions and infrastructure-related characteristics: effective coverage (%) and adjusted odds ratios from logistic regression analyses.

ANC interventions	Effective coverage (%)		Adjusted odds ratio (95% CI)^¥^
	One or more missing infrastructure (n = 728)	Both lab and ultrasound (n = 631)	
**Screening for hypertension**	7	14	2.2 (1.5, 3.1)
**SFH measurement**	7	4	0.6 (0.4, 1.0)
**Screening for anemia**	12	17	1.5 (1.1, 2.1)
**Antenatal ultrasound**	20	36	2.2 (1.7, 2.8)
**Screening for gestational diabetes mellitus**	32	37	1.2 (1.0, 1.5)
**Screening for asymptomatic bacteriuria**	42	43	1.0 (0.8, 1.3)
**Screening for Rh-type**	59	70	1.7 (1.3, 2.1)
**Screening for tetanus immunization status**	37	29	0.7 (0.5, 0.9)

^¥^derived from multivariable logistic regression analyses including all infrastructure-related and maternal sociodemographic variables: laboratory and ultrasound availability, maternal age at pregnancy registration, age at marriage, education and parity

ANC: Antenatal care; SFH: Symphysis-fundal height; CI: Confidence Intervals

A higher proportion of multiparous women (≥four births) had their tetanus immunization checked, compared to nulliparous women (41% vs. 29%; adjusted OR = 2.1, 95% CI: 1.4, 3.2) (S1 Table). None of the other maternal sociodemographic variables had statistically significant associations with effective coverage (S1 Table).

## Discussion

This is the first study to our knowledge to use effective coverage metrics for assessment of the Palestinian health system. By assessing the effective coverage of ANC interventions in public primary healthcare clinics, along with infrastructure-related and maternal sociodemographic factors that may be associated with effective coverage, it was possible to gain insight into ANC service provision in these clinics.

Studies informed by household survey data or direct observations have demonstrated lower effective coverage of ANC than crude service coverage in diverse settings such as Kenya [10], Ethiopia [37] and other countries in sub-Saharan Africa [22]. These studies have assessed the ‘quality’ component of effective coverage using a checklist of services provided during ANC, which would be conceptually equivalent to the outcome ‘coverage of atleast one screening of ANC intervention’ in our study. Almost all pregnant women in our sample had received a blood pressure measurement, and this result was similar to the findings from large multi-country studies of ANC content using survey data [7, 23].

In contrast to other studies of effective coverage that have reported a one-time provision of clinical interventions [10, 22], we also assessed the number and timing of screening tests for the full duration of the pregnancy to produce quality-corrected coverage of ANC interventions using facility-based data. According to outcome definitions used in this study, coverage of at least one screening is not dependant on follow-up care of pregnant women throughout the antenatal period. Coverage of appropriate number of screenings, on the other hand, reflects care provision throughout the antenatal period, but did not factor the timing of screening tests. Effective coverage of ANC interventions is essentially a combination of timely attendance rates and the provision of the prescribed screening test during attendance in the clinics.

Our ANC 4+ coverage rate (60%) was similar to that found in a study using facility-based data conducted in Jordan [38], which has a comparable population and health system as the West Bank. Compared to ANC4+, attendance rates of ANC visits at guideline-specified timings was low in our sample of clinics. As a result, effective coverage of ANC interventions consisting of two-step (screening for gestational diabetes mellitus) or repeat screening tests (screening for anemia and hypertension, SFH measurement, and antenatal ultrasound) were significantly lower than both coverage of atleast one screening and coverage of appropriate number of screenings. A multi-country study reported that 10% of women in Jordan and 27% in Egypt had received a set of routine care components as part of ANC [23]. Despite methodological distinctions in the data source used, this study hints at a trend of low coverage of essential ANC interventions and can corroborate our findings. The difference between coverage of any screening test provided and effective coverage of screening for gestational diabetes (69% vs. 34%) was primarily due to the timing at which the tests were provided.

For ANC interventions consisting of a one-time screening test, the magnitude of the differences between coverage of at least one screening and effective coverage were smaller because timing of provision of ANC interventions played a less decisive role in achieving effective coverage. Indicators of hemoglobin and blood pressure measurement, which are commonly reported worldwide [39], had high coverage of at least one screening but much lower effective coverage in our study.

In general, two underlying contributing factors will lead to low effective coverage of ANC interventions, attendance and service provision. Hijazi et al [38] demonstrated that scheduling of follow-up ANC visits and counseling by care providers were strongly associated with women’s utilization of ANC services in Jordan. Similar explorations are recommended to identify possible issues with providing timely appointments for follow-up ANC visits and potential barriers to ANC utilization in public clinics in the West Bank. Service provision is determined by adherence of care providers to prescribed ANC guidelines, which, in turn, could be influenced by training and supervision, or dissemination of guidelines. Other health systems factors such as lack of supplies of sufficient lab test kits have been shown to be determinants of service delivery in other contexts [19], but is less likely in our setting, considering the relatively high coverage of at least one screening of interventions that need such supplies.

Structural inputs to care such as infrastructure in health facilities have been shown to be weak predictors of content of ANC provided and clinical quality [40], although these results were for countries in sub-Saharan Africa with health systems that may be different from the West Bank. In our study, availability of laboratory and ultrasound in the clinics had varying degrees of associations with effective coverage of the different ANC interventions. A much lower proportion of women had SFH measured in clinics with an ultrasound compared to clinics without, presumably because of the use of antenatal ultrasound for fetal growth monitoring instead. It was beyond the scope of this paper to assess the quality of ultrasound-based fetal growth monitoring. Effective coverage of screening for hypertension and tetanus immunization status, that can be provided to pregnant women without a laboratory or ultrasound in the clinics were still associated with these infrastructure-related variables. Clinics with both a laboratory and ultrasound had a higher effective coverage of hypertension screening due to higher attendance rates in these clinics and relatively routine and non-invasive nature of taking blood pressure. The data available for this study could not shed light on the possible reasons for lower effective coverage of a simple screening test for tetanus immunization status in these better-equipped clinics.

In contrast to infrastructure-related factors, maternal sociodemographic characteristics (maternal age at pregnancy registration, age at marriage, education and parity) were not significantly associated with effective coverage. Differences in effective coverage based on sociodemographic variables may be due to characteristics that were not available for our study. For example, household income or expenditure are commonly used variables for equity analyses, but were not available from the clinical records. Other studies done in LMIC have reported differences in the quality of ANC provided to clients based on their socioeconomic characteristics [16, 41]. These studies used data from household surveys and may have been able to capture populations across social, economic and demographic gradients, compared to our study using only facility-based data of women that receive ANC in public clinics.

In this study, we have presented one approach to the generation of effective coverage using facility-based data. For comprehensive health systems monitoring, such assessments capturing the timing and frequency of care may be used to complement the deficiencies of population-based survey data [23, 42]. Given the availability of routine health facility data from the newly implemented eRegistry in Palestine, health systems monitoring through such metrics is more feasible than with paper-based systems. Inferences derived from our analysis can provide policy-makers with information on some health system factors for consideration to increase effective coverage in public clinics. The eRegistry has incorporated several features designed to increase the level of effective coverage in this population. Specifically, interactive checklists with clinical decision support and automated dashboards providing performance feedback for care providers, can support the provision of complete ANC interventions, while tailored SMS messages to pregnant women, can encourage better uptake of ANC [29].

A limitation of this study was that only documented care was analyzed. Interventions may have been provided without documentation, but for many of these interventions, undocumented screening will be ineffective screening for the purpose of appropriate follow-up during pregnancy. Women may also have received additional targeted tests based on symptoms, as per care providers’ clinical judgements, and subsequently not been re-screened at the time recommended by the guidelines. Such targeted tests may represent reasonable substitutes for routine screening, but would have been missed in our analyses. Effective coverage indicators of screening at specified timings will change over time, as the optimal number and timing of ANC contacts, as well as ANC content, continues to be a matter of debate and subject to evaluation [14, 43–45]. Similar to health systems in other countries in the region [23], pregnant women in the West Bank reportedly seek ANC from private providers and non-governmental organizations, sometimes in addition to receiving ANC from public health facilities. Therefore, the results of this study may not be indicative of the totality of effective coverage of ANC at the population-level in the West Bank, and cannot necessarily be used to estimate how changes in effective coverage in the public health system alone will impact maternal and neonatal health outcomes.

## Conclusion

The choice and definitions of metrics can have substantial impact on health systems monitoring of ANC, both in terms of ascertaining the magnitude of the problem as well as identifying potential solutions. Effective coverage of ANC interventions in public primary healthcare clinics in the West Bank can be increased by improving the timely and complete provision of ANC interventions. Further exploration of specific aspects of care provision in primary healthcare clinics such as care providers’ adherence to guidelines and women’s perceptions and utilization of of ANC services in public clinics, can help address these issues to increase effective coverage of ANC interventions.

## Supporting information

S1 Text ANC interventions in the public health system not included in the analyses. (DOCX)

S2 Text Details of data used in the study. (DOCX)

S1 Table Effective coverage and maternal sociodemographic variables. (DOCX)

## References

[1] Villar J, Bergsjø P. Scientific basis for the content of routine antenatal care I. Philosophy, recent studies, and power to eliminate or alleviate adverse maternal outcomes. Acta Obstetricia et Gynecologica Scandinavica. 1997;76(1):1–14. 10.3109/00016349709047778 9033238

[2] Bhutta ZA, Das JK, Bahl R, Lawn JE, Salam RA, Paul VK, et al Can available interventions end preventable deaths in mothers, newborn babies, and stillbirths, and at what cost? The Lancet. 2014;384(9940):347–70. 10.1016/S0140-6736(14)60792-3 24853604

[3] UNICEF. The State of the World’s Children 2016: A fair chance for every child. June 2016. Available from: https://www.unicef.org/publications/index_91711.html.

[4] WHO. Global Reference List of 100 Core Health Indicators, World Health Organization, Geneva, Switzerland. 2015. Available from: http://www.who.int/healthinfo/indicators/2015/en/. 2015.

[5] Marchant T, Tilley-Gyado RD, Tessema T, Singh K, Gautham M, Umar N, et al Adding Content to Contacts: Measurement of High Quality Contacts for Maternal and Newborn Health in Ethiopia, North East Nigeria, and Uttar Pradesh, India. PloS one. 2015;10(5):e0126840 10.1371/journal.pone.0126840 26000829 PMC4441429

[6] Carvajal-Aguirre L, Amouzou A, Mehra V, Ziqi M, Zaka N, Newby H. Gap between contact and content in maternal and newborn care: An analysis of data from 20 countries in sub-Saharan Africa. Journal of global health. 2017;7(2):020501 10.7189/jogh.07.020501 . 29423178 PMC5804037

[7] Hodgins S, D'Agostino A. The quality–coverage gap in antenatal care: toward better measurement of effective coverage. Global health, science and practice. 2014;2 10.9745/ghsp-d-13-00176 25276575 PMC4168625

[8] Arsenault C, Jordan K, Lee D, Dinsa G, Manzi F, Marchant T, et al Equity in antenatal care quality: an analysis of 91 national household surveys. The Lancet Global Health. 2018;6(11):e1186–e95. 10.1016/S2214-109X(18)30389-9 30322649 PMC6187112

[9] Kyei NNA, Chansa C, Gabrysch S. Quality of antenatal care in Zambia: a national assessment. BMC Pregnancy and Childbirth. 2012;12(1):151 10.1186/1471-2393-12-151 23237601 PMC3536568

[10] Nguhiu PK, Barasa EW, Chuma J. Determining the effective coverage of maternal and child health services in Kenya, using demographic and health survey data sets: tracking progress towards universal health coverage. Tropical Medicine & International Health. 2017;22(4):442–53. 10.1111/tmi.12841 PMC5396138. 28094465 PMC5396138

[11] Austin A, Langer A, Salam RA, Lassi ZS, Das JK, Bhutta ZA. Approaches to improve the quality of maternal and newborn health care: an overview of the evidence. Reproductive health. 2014;11(Suppl 2):S1–S. 10.1186/1742-4755-11-S2-S1 PMC4160919. 25209614 PMC4160919

[12] Villar J, Ba'aqeel H, Piaggio G, Lumbiganon P, Belizán JM, Farnot U, et al WHO antenatal care randomised trial for the evaluation of a new model of routine antenatal care. The Lancet. 2001;357(9268):1551–64. 10.1016/S0140-6736(00)04722-X. 11377642

[13] WHO recommendations on antenatal care for a positive pregnancy experience. 2016. Available from: http://www.who.int/reproductivehealth/publications/maternal_perinatal_health/anc-positive-pregnancy-experience/en/. World Health Organization, Geneva, Switzerland. 28079998

[14] Dowswell T, Carroli G, Duley L, Gates S, Gulmezoglu AM, Khan-Neelofur D, et al Alternative versus standard packages of antenatal care for low-risk pregnancy. Cochrane Database Syst Rev. 2010;(10):CD000934. 10.1002/14651858.CD000934.pub2 20927721 PMC4164448

[15] Bollini P, Quack-Lötscher K. Guidelines-based indicators to measure quality of antenatal care. Journal of Evaluation in Clinical Practice. 2013;19(6):1060–6. 10.1111/jep.12027 23527697

[16] Joshi C, Torvaldsen S, Hodgson R, Hayen A. Factors associated with the use and quality of antenatal care in Nepal: a population-based study using the demographic and health survey data. BMC Pregnancy and Childbirth. 2014;14(1):94 10.1186/1471-2393-14-94 24589139 PMC3943993

[17] WHO. Antenatal care in developing countries: promises, achievements and missed opportunities: an analysis of trends, levels and differentials, 1990–2001. World Health Organization, Geneva, Switzerland; 2003. Available from: http://apps.who.int/iris/bitstream/handle/10665/42784/9241590947.pdf?sequence=1.

[18] Kiwanuka Henriksson D, Fredriksson M, Waiswa P, Selling K, Swartling Peterson S. Bottleneck analysis at district level to illustrate gaps within the district health system in Uganda. Global Health Action. 2017;10(1):1327256 10.1080/16549716.2017.1327256 PMC5496050. 28581379 PMC5496050

[19] Ulrika Baker SP, Tanya Marchant, Godfrey Mbaruku, Silas Temu, Fatuma Manzi & Claudia Hanson Identifying implementation bottlenecks for maternal and newborn health interventions in rural districts of the United Republic of Tanzania. Bulletin of the World Health Organization. 2015;(93):380–9. 10.2471/BLT.14.141879. 26240459 PMC4450702

[20] Tanahashi T. Health service coverage and its evaluation. Bulletin of the World Health Organization. 1978;56(2):295–303. . 96953 PMC2395571

[21] Do M, Micah A, Brondi L, Campbell H, Marchant T, Eisele T, et al Linking household and facility data for better coverage measures in reproductive, maternal, newborn, and child health care: systematic review. Journal of global health. 2016;6(2):020501–. Epub 09/03. 10.7189/jogh.06.020501 . 27606060 PMC5012234

[22] Leslie HH, Malata A, Ndiaye Y, Kruk ME. Effective coverage of primary care services in eight high-mortality countries. BMJ Global Health. 2017;2(3):e000424 10.1136/bmjgh-2017-000424 29632704 PMC5887868

[23] Benova L, Tunçalp Ö, Moran AC, Campbell OMR. Not just a number: examining coverage and content of antenatal care in low-income and middle-income countries. BMJ Global Health. 2018;3(2):e000779 10.1136/bmjgh-2018-000779 29662698 PMC5898334

[24] Kanyangarara M, Munos MK, Walker N. Quality of antenatal care service provision in health facilities across sub–Saharan Africa: Evidence from nationally representative health facility assessments. Journal of global health. 2017;7(2):021101 10.7189/jogh.07.021101 PMC5680531. 29163936 PMC5680531

[25] Giacaman R, Khatib R, Shabaneh L, Ramlawi A, Sabri B, Sabatinelli G, et al Health status and health services in the occupied Palestinian territory. The Lancet. 2009;373(9666):837–49. 10.1016/S0140-6736(09)60107-0

[26] Rahim HFA, Wick L, Halileh S, Hassan-Bitar S, Chekir H, Watt G, et al Maternal and child health in the occupied Palestinian territory. The Lancet. 2009;373(9667):967–77. 10.1016/S0140-6736(09)60108-2 19268353

[27] Ministry of Health, PHIC, Health Status, Palestine, 2016, July 2017. Available from: https://www.site.moh.ps/. Accessed August 2018.

[28] Palestinian Multiple Indicator Cluster Survey 2014, Final Report, Palestinian Central Bureau of Statistics, Ramallah, Palestine. Available from: http://mics.unicef.org/news_entries/32.

[29] Venkateswaran M, Mørkrid K, Ghanem B, Abbas E, Abuward I, Baniode M, et al eRegQual—an electronic health registry with interactive checklists and clinical decision support for improving quality of antenatal care: study protocol for a cluster randomized trial. Trials. 2018;19(1):54 10.1186/s13063-017-2386-5 29357912 PMC5778657

[30] Venkateswaran M, Mørkrid K, Abu Khader K, Awwad T, Friberg IK, Ghanem B, et al Comparing individual-level clinical data from antenatal records with routine health information systems indicators for antenatal care in the West Bank: A cross-sectional study. PloS one. 2018;13(11):e0207813 10.1371/journal.pone.0207813 30481201 PMC6258527

[31] Dean AG, Sullivan KM, Soe MM. OpenEpi: Open Source Epidemiologic Statistics for Public Health. Available from: http://www.openepi.com/Menu/OE_Menu.htm.

[32] Bell BA, Onwuegbuzie AJ, Ferron JM, Jiao QG, Hibbard ST, Kromrey JD. Use of Design Effects and Sample Weights in Complex Health Survey Data: A Review of Published Articles Using Data From 3 Commonly Used Adolescent Health Surveys. American Journal of Public Health. 2012;102(7):1399–405. 10.2105/AJPH.2011.300398 PMC3477989. 22676502 PMC3477989

[33] Health Information Systems Programme (HISP). District Health Information System 2 (DHIS 2). Available from: https://www.dhis2.org/.

[34] Harmonized Reproductive Health eRegistry, Palestinian National Institute of Public Health. Available from: www.pniph.org. Accessed January 2019.

[35] WHO. Essential Interventions, Commodities and Guidelines for Reproductive, Maternal, Newborn and Child Health. 2012. Available from: http://www.who.int/pmnch/knowledge/publications/201112_essential_interventions/en/. World Health Organization, Geneva, Switzerland.

[36] StataCorp., svy estimation—Estimation commands for survey data. Available from: https://www.stata.com/manuals13/svysvyestimation.pdf.

[37] Yakob B, Gage A, Nigatu TG, Hurlburt S, Hagos S, Dinsa G, et al Low effective coverage of family planning and antenatal care services in Ethiopia. International Journal for Quality in Health Care. 2019:mzy251-mzy. 10.1093/intqhc/mzy251 30608585

[38] Hijazi HH, Alyahya MS, Sindiani AM, Saqan RS, Okour AM. Determinants of antenatal care attendance among women residing in highly disadvantaged communities in northern Jordan: a cross-sectional study. Reproductive health. 2018;15(1):106–. 10.1186/s12978-018-0542-3 . 29879992 PMC5992715

[39] Morón-Duarte LS, Ramirez Varela A, Segura O, Freitas da Silveira M. Quality assessment indicators in antenatal care worldwide: a systematic review. International Journal for Quality in Health Care. 2018:mzy206-mzy. 10.1093/intqhc/mzy206 30295805

[40] Leslie HH, Sun Z, Kruk ME. Association between infrastructure and observed quality of care in 4 healthcare services: A cross-sectional study of 4,300 facilities in 8 countries. PLOS Medicine. 2017;14(12):e1002464 10.1371/journal.pmed.1002464 29232377 PMC5726617

[41] Victora CG, Matijasevich A, Silveira MF, Santos IS, Barros AJD, Barros FC. Socio-economic and ethnic group inequities in antenatal care quality in the public and private sector in Brazil. Health policy and planning. 2010;25(4):253–61. 10.1093/heapol/czp065 20123940 PMC2889278

[42] Munos MK, Stanton CK, Bryce J, the Core Group for Improving Coverage Measurement for M. Improving coverage measurement for reproductive, maternal, neonatal and child health: gaps and opportunities. Journal of global health. 2017;7(1):010801 10.7189/jogh.07.010801 PMC5460400. 28607675 PMC5460400

[43] Hall MH. Rationalisation of antenatal care. The Lancet. 2001;357(9268):1546 10.1016/S0140-6736(00)04777-2. 11377637

[44] Carroli G, Villar J, Piaggio G, Khan-Neelofur D, Gülmezoglu M, Mugford M, et al WHO systematic review of randomised controlled trials of routine antenatal care. The Lancet. 2001;357(9268):1565–70. 10.1016/S0140-6736(00)04723-1 11377643

[45] Fernandez Turienzo C, Sandall J, Peacock JL. Models of antenatal care to reduce and prevent preterm birth: a systematic review and meta-analysis. BMJ Open. 2016;6(1). 10.1136/bmjopen-2015-009044 26758257 PMC4716175

