# Supplementary material for: Effective coverage of essential antenatal care interventions: A cross-sectional study of public primary healthcare clinics in the West Bank
Source: PLoS One. 2019 Feb 22;14(2):e0212635. doi: 10.1371/journal.pone.0212635 (PMC6386267; doi:10.1371/journal.pone.0212635)
Supplement: S1 Text — (DOCX) [file pone.0212635.s001.docx]

# S1 Text

# ANC interventions in the public health system not included in the analyses

- Use of antihypertensive drugs for treating severe hypertension in pregnancy
- Prevention and treatment of Eclampsia
- Management of prelabour rupture of membranes and preterm labour
- Induction of labour for management of prelabour rupture of membranes at term
- Antibiotics for management of preterm rupture of membranes
- Corticosteroids for prevention of neonatal respiratory distress syndrome
- Management of Vaginal bleeding
