# Supplementary material for: Effective coverage of essential antenatal care interventions: A cross-sectional study of public primary healthcare clinics in the West Bank
Source: PLoS One. 2019 Feb 22;14(2):e0212635. doi: 10.1371/journal.pone.0212635 (PMC6386267; doi:10.1371/journal.pone.0212635)
Supplement: S2 Text — (DOCX) [file pone.0212635.s002.docx]

# S2 Text: Details of data used in the study

## Data source

Paper-based clinical records from 17 primary healthcare clinics included in the data collection of the eRegistry project; clinical records were opened in the clinic in the year 2015 (Jan 1st 2015- Dec 31st 2015).

## List of anonymized and aggregated secondary data obtained for analysis

- Clinics identified by codes
- Availability of lab, ultrasound
- Number of doctors, nurses, midwives, non-nurse/midwife health workers
- Demographic data: Mother’s education in years, age group, age at marriage, age at first pregnancy
- History of: type 1 insulin dependent diabetes mellitus, type 2 diabetes mellitus, gestational diabetes mellitus, anemia, previous C-section (>/=2), previous perinatal deaths (>/=1), previous stillbirths
- Risks in the current pregnancy and reasons for referral
- Gestational ages at visits; at lab tests; at ultrasound examinations
- Content of antenatal care visits:
  - Urine sugar tested at visits- yes or no; Urine sugars at visit- positive or negative
  - Fundal height measurement at visits- yes or no; Fundal height value 1
  - Presentation at visits- yes or no; Presentation 1
  - Iron, folic acid supplementation visits- given or not given
  - Blood pressure values
  - Hemoglobin and hematocrit values
  - Blood sugar and oral glucose tolerance test values
  - Tetanus toxoid vaccination

## Data owned by

Palestinian Ministry of Health

## Contact details for requesting data

Palestinian National Institute of Public Health
Website: [www.pniph.org](http://www.pniph.org)
