# Supplementary material for: Effective coverage of essential antenatal care interventions: A cross-sectional study of public primary healthcare clinics in the West Bank
Source: PLoS One. 2019 Feb 22;14(2):e0212635. doi: 10.1371/journal.pone.0212635 (PMC6386267; doi:10.1371/journal.pone.0212635)
Supplement: S1 Table — (DOCX) [file pone.0212635.s003.docx]

# S1 Table

# Effective coverage and maternal sociodemographic variables

## Table A: Effective coverage (%) of essential ANC interventions across sub-groups based on maternal socioeconomic variables

| Maternal sociodemographic variables | Effective coverage (%, 95% CI) | | | | | | | |
| --- | --- | --- | --- | --- | --- | --- | --- | --- |
|  | Screening for hypertension | SFH measurement | Screening for anemia | Antenatal ultrasound | Screening for tetanus immunization status | Screening for asymptomatic bacteriuria | Screening for Rh-type | Screening for gestational diabetes mellitus |
| Age (years) |  |  |  |  |  |  |  |  |
| <=20 | 8 | 5 | 15 | 27 | 32 | 48 | 71 | 33 |
| 21-34 | 11 | 5 | 14 | 27 | 33 | 41 | 63 | 35 |
| >=35 | 12 | 6 | 16 | 30 | 38 | 42 | 61 | 38 |
| Education (years) |  |  |  |  |  |  |  |  |
| <10 | 10 | 5 | 12 | 30 | 34 | 41 | 67 | 37 |
| 10-13 | 10 | 6 | 16 | 27 | 34 | 42 | 68 | 37 |
| >13 | 11 | 5 | 13 | 27 | 32 | 43 | 60 | 32 |
| Age at marriage (years) |  |  |  |  |  |  |  |  |
| <20 | 11 | 5 | 14 | 27 | 33 | 44 | 67 | 35 |
| >20 | 10 | 6 | 15 | 28 | 33 | 41 | 60 | 34 |
| Parity |  |  |  |  |  |  |  |  |
| 0 | 10 | 6 | 15 | 27 | 29 | 44 | 67 | 33 |
| 1 – 4 | 10 | 5 | 14 | 26 | 35 | 41 | 62 | 36 |
| ≥4 | 13 | 6 | 12 | 33 | 41 | 44 | 62 | 35 |

CI: Confidence Intervals; SFH: Symphysis-fundal height; ANC: Antenatal Care

## Table B: Associations of effective coverage and maternal sociodemographic variables: adjusted odds ratios and 95% CI

| Maternal sociodemographic variables | Adjusted OR (95% CI) ^¥^ | | | | | | | |
| --- | --- | --- | --- | --- | --- | --- | --- | --- |
|  | Screening for hypertension | SFH measurement | Screening for anemia | Antenatal ultrasound | Screening for tetanus immunization status | Screening for asymptomatic bacteriuria | Screening for Rh-type | Screening for gestational diabetes mellitus |
| Age (years) |  |  |  |  |  |  |  |  |
| <=20 | 1 | 1 | 1 | 1 | 1 | 1 | 1 | 1 |
| 21-34 | 1.7 (0.9,3.3) | 1.1 (0.5,2.4) | 1.0 (0.6,1.6) | 0.9 (0.6,1.4) | 0.8 (0.5,1.1) | 0.8 (0.6,1.1) | 0.9 (0.6,1.3) | 1.1 (0.8,1.6) |
| >=35 | 1.8 (0.7,4.5) | 1.1 (0.4,3.7) | 1.3 (0.6,2.8) | 0.8 (0.4,1.5) | 0.8 (0.4,1.4) | 0.8 (0.5,1.4) | 0.8 (0.4,1.4) | 1.3 (0.7,2.3) |
| Education (years) |  |  |  |  |  |  |  |  |
| <10 | 1 | 1 | 1 | 1 | 1 | 1 | 1 | 1 |
| 10-13 | 0.9 (0.5,1.8) | 1.2 (0.5,2.7) | 1.3 (0.8,2.3) | 0.9 (0.6,1.3) | 1.2 (0.8,1.8) | 1.0 (0.7,1.5) | 0.9 (0.6,1.3) | 1.0 (0.7,1.5) |
| >13 | 1.1 (0.6,2.2) | 0.9 (0.4,2.2) | 0.9 (0.5,1.7) | 0.8 (0.5,1.2) | 1.1 (0.7,1.7) | 1.3 (0.8,1.7) | 0.7 (0.4,1.0) | 0.8 (0.5,1.2) |
| Age at marriage (years) |  |  |  |  |  |  |  |  |
| <20 | 1 | 1 | 1 | 1 | 1 | 1 | 1 | 1 |
| >20 | 0.7 (0.5,1.1) | 1.2 (0.7,2.2) | 1.2 (0.8,1.7) | 1.2 (0.9,1.7) | 1.2 (0.9,1.7) | 0.9 (0.7,1.2) | 0.8 (0.6,1.1) | 1.0 (0.8,1.4) |
| Parity |  |  |  |  |  |  |  |  |
| 0 | 1 | 1 | 1 | 1 | 1 | 1 | 1 | 1 |
| 1 – 4 | 0.8 (0.5,1.2) | 0.7 (0.4,1.3) | 1.0 (0.7,1.4) | 1.1 (0.8,1.4) | 1.4 (1.0,2.0) | 0.9 (0.7,1.2) | 0.8 (0.6,1.0) | 1.1 (0.8,1.4) |
| ≥4 | 1.0 (0.5,2.0) | 0.9 (0.4,2.2) | 0.7 (0.4,1.4) | 1.4 (0.9,2.2) | 2.1 (1.4,3.2) | 1.1 (0.7,1.6) | 0.7 (0.5,1.1) | 0.9 (0.6,1.4) |

^¥^derived from multivariable logistic regression analyses including all infrastructure-related and maternal sociodemographic variables: laboratory and ultrasound availability, maternal age at pregnancy registration, age at marriage, education and parity; CI: confidence intervals; SFH: symphysis-fundus height
